# Divergent Risk Factors and Outcomes in Hemolysis, Elevated Liver Enzymes, and Low Platelets Syndrome and Isolated Preeclampsia

**DOI:** 10.1016/j.ekir.2026.106681

**Published:** 2026-06-22

**Authors:** Jean-Michel Halimi, Grégory Lailler, Valentin Maisons, Bénédicte Sautenet, Laurent Fauchier, Jean-Baptiste de Fréminville, Jacques Blacher, Valérie Olié

**Affiliations:** 1 Service de Néphrologie-hypertension, Dialyses, Transplantation Rénale, Hôpital Bretonneau, Tours, France; 2 Institut National de la Santé et de la Recherche Médicale (INSERM) U1327, Université de Tours, Tours, France; 3 Santé publique France, Saint-Maurice, France; 4 Institut National de la Santé et de la Recherche Médicale U1246, SPHERE, Tours, France; 5 Service de Cardiologie, Hôpital Trousseau, Tours, France; 6 Service de Médecine vasculaire, Hôpital Trousseau, France; 7 Centre de diagnostic et de thérapeutique, Hôtel Dieu, Assistance Publique – Hôpitaux de Paris, Université Paris Cité, Paris, France

**Keywords:** epidemiology, pregnancy, thrombotic microangiopathy

## Abstract

**Introduction:**

The hemolysis, elevated liver enzymes, and low platelets (HELLP) syndrome usually occurs in association with preeclampsia (PE). Risk factors and short-term and long-term outcomes of women with PE with HELLP (PE-HELLP) or isolated PE (iPE) are unclear.

**Methods:**

Using data from the Cohort of Cardiovascular Diseases in Pregnancy (CONCEPTION) (a French nationwide, population-based, prospective cohort) study, all women who gave birth at ≥22 weeks of gestation between 2010 and 2018 were included. The main outcomes were mortality, cardiovascular, cerebrovascular, thromboembolic events, and renal failure (RF).

**Results:**

Among the 2,829,700 women with first deliveries, 73,736 women with iPE, 9147 women with PE-HELLP and 2,746,817 women controls were studied. Diabetes, chronic hypertension, and obesity were more frequently associated with iPE than PE-HELLP. Social deprivation was positively associated with iPE but negatively associated with PE-HELLP. Chronic inflammatory diseases and lupus were more associated with PE-HELLP than iPE. During the first year, iPE and PE-HELLP constituted major risk factors for hypertension and all other major adverse events versus controls; however, PE-HELLP was a significantly stronger risk of major adverse cardiovascular events (MACEs), stroke, thromboembolism, and RF than iPE. After the first year, iPE remained significantly associated with all outcomes, whereas PE-HELLP was a risk factor only for hypertension and RF versus controls; the risk of RF was similar for iPE and PE-HELLP.

**Conclusion:**

iPE and PE with HELLP differ regarding their risk factors and types and dynamics of their major complications.

## Graphical abstract

**Figure:**
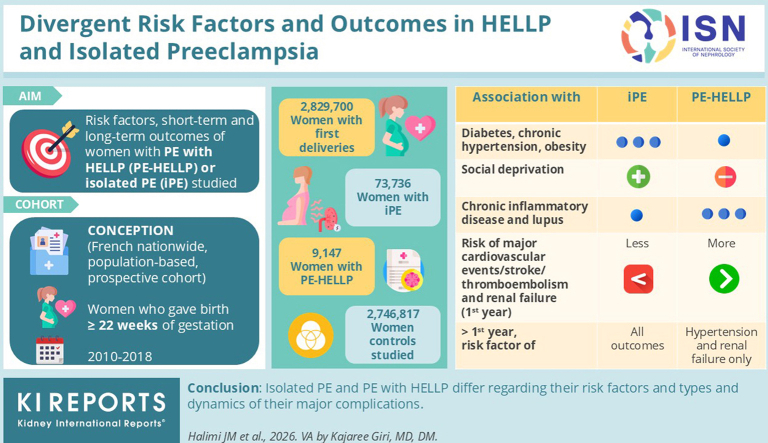

The HELLP syndrome is a rare but severe pregnancy complication, occurring in approximately 0.5%–1% of pregnancies.^1^ HELLP syndrome usually occurs in association with PE, and for this reason HELLP-PE is usually considered as the same entity. However, whether it represents a distinct condition or a severe variant within the PE spectrum has been debated.^2^ HELLP syndrome may differ from PE to a large extent.^2^ Whereas iPE is more prevalent and more likely characterized by high blood pressure and proteinuria, HELLP syndrome is associated with a higher burden of multiorgan involvement, including acute kidney injury and severe hematological and hepatic conditions.^3^ Furthermore, HELLP syndrome is characterized by its specific biological presentation and the involvement of the placenta-liver axis with liver thrombotic microangiopathy due to severe liver endothelial damage.^4^^,^^5^

Although the short-term cardiovascular and renal outcomes after PE have been described,^6^ data comparing outcomes and their dynamics according to the presence or absence of HELLP syndrome remain limited.

Using data from the nationwide French CONCEPTION study derived from the French National Health Data System (Système National des Données de Santé [SNDS]), the present study aimed to: (i) assess short-term (< 1 year) and medium-term (≥ 1 year) risks of mortality, RF, and cardiovascular outcomes among women with iPE and those with PE-HELLP, compared with women with normotensive pregnancies; and (ii) compare these risks between iPE and PE-HELLP.

## Methods

### Data Source

The CONCEPTION study is a nationwide, population-based, prospective cohort study aimed at investigating the epidemiology of hypertensive disorders and cardiovascular events among women who gave birth in France at ≥22 weeks of gestation between January 1, 2010, and December 31, 2018. The study includes data for >4.5 million women and 7.1 million childbirths all sourced from the SNDS. The SNDS database provides comprehensive information on all health care expenditure reimbursed by the national health insurance system for the entire French population (∼ 66 million individuals). It integrates 2 main data sources, namely, the National Hospital Discharge Database, which captures details of public and private hospital stays, including diagnoses coded using the International Classification of Diseases, Tenth Revision (ICD-10); and the Interscheme Consumption Data Mart, which records out-of-hospital care such as medications, imaging, and outpatient medical services.

In accordance with French national regulations and ethics committee requirements, neither participant consent nor institutional review board approval was required for this study. Santé Publique France, the French public health agency, holds permanent and full access to the SNDS (governmental deliberation no. 2016-316, dated October 13, 2016). Because of regulatory restrictions, SNDS data cannot be shared and are accessible only through a secure portal, for which specific registration and regulatory clearance are mandatory.

### Study Population

For the present study, we included all women in the CONCEPTION study who gave birth for the first time in France after 22 weeks of gestation between January 1, 2010, and December 31, 2018. Before 22 weeks of gestation, miscarriages are not systematically documented in medical records in France; thus, their exclusion from the present analyses. For women who gave birth by vaginal delivery, the parity was directly available in patients’ medical records. Women who had a cesarean delivery were considered primiparous if no previous childbirth was identified since 2006. We excluded women aged > 49 years, and those with an invalid date of pregnancy. Study follow-up spanned from the beginning of the first pregnancy to the beginning of the second pregnancy, death, or December 31, 2021, whichever came first.

### PE and HELLP Syndrome

For all women included in the final population, we identified all cases of iPE and PE-HELLP during the first pregnancy until December 31, 2021, with algorithms based on diagnoses coded during hospital stays (ICD-10). PE was defined as hospitalization with ICD-10 diagnosis codes O11, O14, or O15. It was dichotomized into iPE or PE-HELLP according to the presence of an ICD-10 code of HELLP syndrome (O14.2). Because HELLP syndrome is a subcode of PE in the ICD-10 classification, it was not possible to consider the entity HELLP without PE, even though HELLP and PE may be considered distinct conditions. Therefore, the exposure was treated as a 3-level categorical variable: no PE, PE-HELLP, and iPE. PE was defined as early onset when it occurred before 34 weeks of gestation, and late onset otherwise. For singleton pregnancies with a delivery in or after 2013, PE combined with small-for-gestational-age infant was defined as a birth weight below the 10th percentile for gestational age and sex.^7^ This year was chosen because data on the fetus’ weight and sex before 2013 were not exhaustive (Supplementary Table S1: ICD codes).

### Outcomes

The study outcomes were mortality; the occurrence of definitive postpregnancy hypertension and diabetes; and the following cardiovascular, cerebrovascular, and renal events during follow-up: stroke, acute coronary syndrome, heart failure, venous thromboembolism, pulmonary embolism, and chronic kidney disease (CKD). New-onset chronic hypertension or diabetes during the study follow-up were defined as receiving prescribed antihypertensive or antidiabetic drugs, respectively, on ≥3 different dates (2 different dates if ≥1 large package of 90 pills was dispensed) over a rolling period of 1 year. We considered the date of the first dispensing of antihypertensive or antidiabetic drugs as the date of diagnosis of chronic hypertension or diabetes, respectively. Women diagnosed with one of these conditions before or during the study were considered to have it for the rest of the follow-up period, regardless of whether antihypertensive or antidiabetic drugs treatment was interrupted.

RF was defined either by ICD-10 codes or by the identification of ≥40 dialysis sessions during the follow-up for chronic dialysis (this figure was important to differentiate chronic from acute dialysis). Other events, except mortality, were identified by the primary diagnoses coded during hospitalization (Supplementary Table S1). The date of hospitalization was considered the date of the event. With regard to mortality, the date of death was directly available in the SNDS. MACE was defined as a composite outcome combining stroke, acute coronary syndrome, heart failure, and death.

### Baseline Characteristics and Covariates

Preexisting chronic hypertension or diabetes were identified by the dispensing of 3 antihypertensive or antidiabetic drugs, respectively, on 3 different dates in the year preceding the first pregnancy, or on 2 dates if ≥1 large package (i.e., 90 pills) was dispensed. Women were defined as having preexisting chronic hypertension if they were hospitalized with a primary diagnosis of preexisting chronic hypertension (ICD-10 codes: O10, O11) during pregnancy or postpartum. Obesity and tobacco smoking were identified by ICD-10 codes during hospitalization, and by the dispensing of prescribed nicotine replacement therapy before or during pregnancy for tobacco smoking. People who benefited from Universal Medical Coverage, a social benefit in France for those whose income is below a certain ceiling, at baseline were defined as living in social deprivation. A medical history of cardiovascular, cerebrovascular, renal, or chronic inflammatory disease; systemic lupus erythematosus; or HIV was identified through diagnoses coded during hospital stays or through recognition of long-term disease, since 2006. The characteristics of pregnancy management were identified in the medical record: length of stay, red blood cells or blood products transfusion, patient transfer to a resuscitation unit, in-hospital mortality.

### Statistical Analysis

We first described the baseline characteristics (number and % for categorical variables, means and SDs for continuous variables) for all women combined, and then for those who had a PE-HELLP, or iPE, or those without PE. We then calculated the rates of each outcome during follow-up for the same subpopulations.

We used multinomial logistic regression models to estimate the crude and fully adjusted odds ratios (ORs) and 95% confidence interval of iPE and PE-HELLP during the first pregnancy according to antenatal maternal characteristics (age, hypertension, diabetes, tobacco use, obesity, social deprivation, CKD, congenital heart defect, chronic inflammatory disease, systemic lupus erythematosus, HIV status). In all models, the outcome was treated as a 3-level variable: no PE (reference), iPE, PE-HELLP.

The associations between iPE or PE-HELLP and the onset of the outcomes during follow-up were assessed using hazard ratios (HRs) calculated using crude and adjusted Cox proportional regression models. A first set of models studied the occurrence of the outcomes during the first year after childbirth, starting from the end of the postpartum period (6 weeks after childbirth). A second set of models studied the occurrence of the outcomes from the end of the first year after childbirth to the end of the follow-up. The follow-up was censored at the beginning of second pregnancy, death, or December 31, 2021, whichever came first. Models were adjusted on tobacco smoking, obesity, social deprivation, and history of diabetes.

Maternal age in years was used as the time scale instead of time-on-study in all Cox models, because this method reduces age-related confounding more efficiently than subsequently adjustment or stratification for maternal age.^8^ The models were adjusted for social deprivation, obesity, tobacco smoking, and history of diabetes. All statistical analyses were conducted using SAS Guide 8 (version 9.4; SAS Institute, Carry, NC).

## Results

### Baseline Characteristics

**Figure 1:**
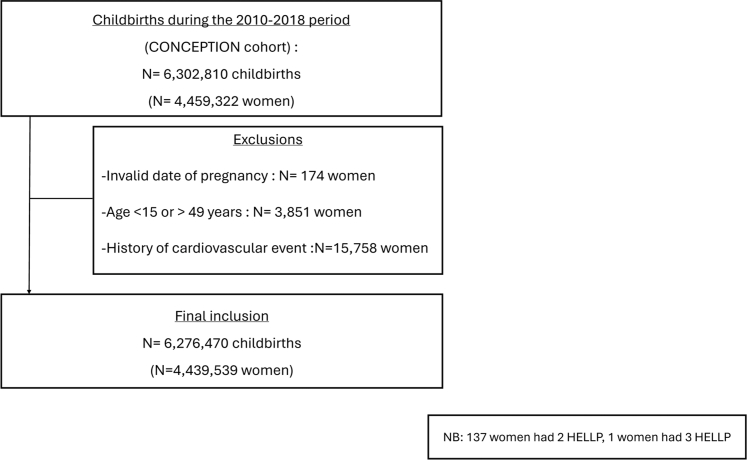
Flow chart. Selection of women with preeclampsia with or without HELLP. HELLP, hemolysis, elevated liver enzymes, and low platelets syndrome.

After excluding 3851 women aged < 15 years or > 49 years, and 174 with an invalid date of pregnancy, a total of 2,829,700 women with first deliveries in France between 2010 and 2018 were included in this analysis (Figure 1). Among them, 73,736 had an iPE and 9147 had a PE-HELLP. The characteristics of the study population according to the occurrence of iPE or PE-HELLP are displayed in Table 1. Women who had an iPE or a PE-HELLP were on average aged > 1 year than controls. They were more likely to have a first multiple pregnancy, to live in deprivation, to have a history of chronic hypertension, diabetes, obesity, venous thromboembolism, CKD, chronic inflammatory disease, or HIV infection (Table 1).

**Table 1:** Characteristics at the beginning of the first pregnancy, according to the occurrence of a PE with or without HELLP

Parameters	Total		PE without HELLP		PE-HELLP		No PE	
	(*N* = 2,829,700)		(*n* = 73,736)		(*n* = 9147)		(*n* = 2,746,817)	
	Mean	SD	Mean	SD	Mean	SD	Mean	SD
Age (yrs)	28.3	5.36	29.43	6.06	29.81	5.58	28.27	5.34
Mean length of stay (d)	9.92	11.86	13.55	9.15	12.1	8.74	9.4	12.16
	*N*	%	*n*	%	*n*	%	*n*	%
Multiple pregnancy	60,820	2.15	6,083	8.25	842	9.21	53,895	1.96
Social deprivation	387,935	13.71	12,422	16.85	1075	11.75	374,438	13.63
Obesity	117,300	4.15	8,807	11.94	803	8.78	107,69	3.92
Chronic hypertension	43,088	1.52	6643	9.01	695	7.6	35,750	1.3
Diabetes	14,642	0.52	1545	2.1	95	1.04	13,002	0.47
Tobacco use	252,353	8.92	6475	8.78	648	7.08	245,23	8.93
Medical history								
Heart failure	235	0.01	25	0.03	.	.	210	0.01
Stroke	1749	0.06	72	0.1	5	0.05	1672	0.06
Veinous thromboembolism	4310	0.15	192	0.26	37	0.4	4081	0.15
Chronic kidney disease	1235	0.04	257	0.35	18	0.2	960	0.03
Heart defect	2848	0.1	103	0.14	17	0.19	2728	0.1
Chronic inflammatory disease	27,882	0.99	1043	1.41	178	1.95	26,661	0.97
Systemic lupus erythematosus	3027	0.11	172	0.23	42	0.46	2813	0.1
HIV	3186	0.11	178	0.24	22	0.24	2986	0.11

HELLP, hemolysis, elevated liver enzymes, and low platelets syndrome; PE, preeclampsia.

### Mother and Child Complications

The median gestational age was 39 weeks for women who did not have PE, 37 weeks for women who had iPE, and 35 weeks for women who had PE-HELLP (Table 2). Among women who had PE-HELLP, 80.54% delivered via caesarean delivery, 17.7% were transferred to an intensive care unit, 9.1% had a red blood cells transfusion, 7.7% had a blood product transfusion, and 0.1% died in hospital (Table 2). Fetal complications were more likely after PE-HELLP than after iPE. After PE-HELLP, 51.2% of children were small for gestational age, 64.5% were preterm, and 0.4% died *in utero* or after birth. Among women who had PE-HELLP, 10.5% had gestational diabetes, 45.1% had an early PE, 6.5% had an eclampsia, 15.9 had an obstetric hemorrhage, 0.4% had a venous thromboembolism, 0.3% had a stroke, and 0.1% died.

**Table 2:** Mother and child complications according to the occurrence of a PE with or without HELLP

Parameters	Total		PE without HELLP		PE-HELLP		No PE	
	(*N* = 2,829,700)		(*N* = 73,736)		(*N* = 9147)		(*N* = 2,746,817)	
	Mean or median	SD	Mean or median	SD	Mean or median	SD	Mean or median	SD
Gestational age (WG): mean	38.94	2.26	36.41	3.43	34.19	4.28	39.03	2.15
Gestational age (WG): median	39	2.26	37	3.43	35	4.28	39	2.15
	*N*	%	*n*	%	*n*	%	*n*	%
Child complications								
Infant death	1240	0.04	66	0.09	33	0.36	1141	0.04
Small for gestational age^a^	235,411	14.21	13,945	35.98	2675	51.23	218,791	13.56
Preterm birth	229,200	8.1	30,087	40.8	5895	64.45	193,218	7.03
Moderate preterm (≥ 32 WG)	30,370	1.07	5943	8.06	1778	19.44	22,649	0.82
Very preterm (27–31 WG)	18,223	0.64	1164	1.58	565	6.18	16,494	0.6
Extremely preterm (< 27 WG)	180,607	6.38	22,980	31.17	3552	38.83	15,4075	5.61
Mother complications								
Gestational diabetes	237,089	8.38	9632	13.06	964	10.54	226,493	8.25
Early preeclampsia (< 34 WG)	23,509	0.83	19,386	26.29	4123	45.07	.	.
Eclampsia	3337	0.12	2741	3.72	596	6.52	.	.
Obstetric hemorrhage	162,187	5.73	6746	9.15	1454	15.9	153,987	5.61
Veinous thromboembolism	3035	0.11	148	0.2	35	0.38	2852	0.1
Acute coronary syndrome	84	0	6	0.01	4	0.04	74	0
Stroke (all types)	513	0.02	65	0.09	26	0.28	422	0.02
Ischemic stroke	255	0.01	28	0.04	8	0.09	219	0.01
Hemorrhage stroke	268	0.01	40	0.05	19	0.21	209	0.01
In-hospital death	196	0.01	10	0.01	9	0.1	177	0.01
Management during childbirth								
Cesarean delivery	758,434	26.8	46,084	62.5	7367	80.54	704,983	25.67
Transfer to a resuscitation unit	7445	0.26	1846	2.5	1621	17.72	3978	0.14
RBC transfusion	19,012	0.67	1597	2.17	831	9.08	16,584	0.6
Blood products transfusion	6132	0.22	607	0.82	703	7.69	4822	0.18

HELLP, hemolysis, elevated liver enzymes, and low platelets syndrome; PE, preeclampsia; RBC, red blood cells; WG, weeks of gestation.

a Birth weight was available from 2013; percentages for small-gestational-age were calculated for the period from 2013 to 2018 (*n* = 1,657,104).

### Risk of Developing iPE or PE-HELLP

In Table 3, we present the crude OR and adjusted OR (aOR) of developing an iPE or a PE-HELLP according to maternal characteristics and medical history. In the fully adjusted model, maternal age > 30 years, chronic hypertension, diabetes, obesity, CKD, chronic inflammatory diseases, and systemic lupus erythematosus were significantly associated with both iPE or PE-HELLP. Tobacco smoking was negatively associated with iPE or PE-HELLP. Social deprivation was associated with iPE (aOR [95% confidence interval]: 1.29 [1.27–1.32]) but negatively associated with PE-HELLP (aOR = 0.92 [0.86–0.99]). Chronic hypertension was more strongly associated with iPE (aOR = 5.62 [5.46–5.78]) than with PE-HELLP (aOR = 5.00 [4.61–5.42]). Conversely, chronic inflammatory diseases and systemic lupus erythematosus were more associated with PE-HELLP than with iPE.

**Table 3:** Crude and adjusted odds ratios of developing preeclampsia with or without HELLP syndrome during the first pregnancy according to women’s characteristics and medical history

Parameters	Crude odds ratios		Adjusted odds ratios	
	PE without HELLP	PE-HELLP	PE without HELLP	PE-HELLP
Age category				
< 20 yr	0.95 (0.91–0.99)	0.62 (0.54–0.72)	0.87 (0.84–0.91)	0.68 (0.59–0.78)
20–30 yr	Ref	Ref	Ref	Ref
30–40 yr	1.26 (1.24–1.28)	1.45 (1.39–1.51)	1.21 (1.19–1.23)	1.38 (1.32–1.44)
> 40 yr	2.64 (2.56–2.73)	2.39 (2.18–2.62)	2.13 (2.06–2.20)	2.03 (1.85–2.23)
Chronic hypertension	7.51 (7.31–7.72)	6.24 (5.77–6.74)	5.62 (5.46–5.78)	5.00 (4.61–5.42)
Diabetes	4.51 (4.27–4.75)	2.21 (1.80–2.70)	2.34 (2.21–2.48)	1.34 (1.09–1.65)
Tobacco use	0.98 (0.96–1.01)	0.78 (0.72–0.84)	0.90 (0.87–0.92)	0.74 (0.69–0.81)
Obesity	3.32 (3.25–3.40)	2.36 (2.19–2.54)	2.75 (2.69–2.82)	2.11 (1.96–2.27)
Social deprivation	1.28 (1.26–1.31)	0.84 (0.79–0.90)	1.29 (1.27–1.32)	0.92 (0.86–0.99)
Chronic kidney disease	10.00 (8.72–11.48)	5.70 (3.58–9.07)	2.73 (2.33–3.18)	1.81 (1.13–2.93)
Congenital heart defect	1.41 (1.16–1.71)	1.87 (1.16–3.02)	0.98 (0.80–1.20)	1.43 (0.89–2.31)
Chronic inflammatory disease	1.33 (1.24–1.43)	1.80 (1.53–2.13)	1.14 (1.06–1.23)	1.52 (1.26–1.82)
Systemic lupus erythematosus	2.28 (1.96–2.66)	4.53 (3.34–6.14)	1.23 (1.03–1.47)	2.01 (1.41–2.86)
HIV infection	2.23 (1.91–2.59)	2.22 (1.46–3.38)	1.31 (1.10–1.56)	1.10 (0.70–1.74)

HELLP, hemolysis, elevated liver enzymes, and low platelets syndrome; PE, preeclampsia.

### Major Outcomes Within the First Year Following Pregnancy

**Figure 2:**
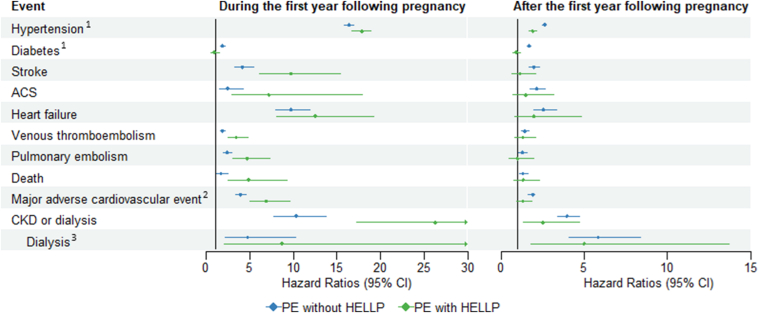
Adjusted hazard ratios of developing hypertension, diabetes, or cardiovascular diseases within and after the first year following a preeclampsia with or without HELLP syndrome (forest plot). ^1^The models estimating the hazard ratios of developing hypertension and diabetes were performed in the subpopulations of women without history of hypertension (*n* = 2,786,612) or diabetes (*n* = 2,815,058), respectively. ^2^These models were adjusted on tobacco smoking, obesity, social deprivation, and history of diabetes. ^3^At least 40 dialysis sessions (to ensure that it corresponds to chronic, not acute dialysis). ACS, acute coronary syndrome; CI, confidence interval; CKD, chronic kidney disease; HELLP, hemolysis, elevated liver enzymes, and low platelets syndrome; PE, preeclampsia. Control group: pregnancies without HELLP or preeclampsia. Major adverse cardiovascular events include stroke, ACS, heart failure, and death.

During the first year following pregnancy, women with iPE or those with PE-HELLP had a higher risk of chronic hypertension, stroke, acute coronary syndrome, heart failure, venous thromboembolism, death, CKD, or chronic dialysis than women who had a pregnancy without PE, both in univariate analyses and after multiple adjustments (Table 4). iPE was associated with a higher risk of diabetes (adjusted HR [aHR] = 1.87 [1.66–2.11]) but not PE-HELLP (aHR = 0.90 [0.55–1.47]). The risk of MACE (stroke, acute coronary syndrome, heart failure, or death) was greater in women with iPE or PE-HELLP versus controls but it was stronger for PE-HELLP (aHR = 6.90 [4.98–9.62]) than for iPE (aHR = 3.91 [3.32–4.59]). Similar results were found for the risk of stroke, venous thromboembolism (including pulmonary embolism) and CKD (Table 4, Figure 2).

**Table 4:** Crude and adjusted hazard ratios of developing hypertension, diabetes or cardiovascular diseases within the first year following a preeclampsia with or without HELLP syndrome

Parameters	Crude hazard ratios			Adjusted hazard ratios^a^		
	No PE	PE without HELLP	PE-HELLP	No PE	PE without HELLP	PE-HELLP
Hypertension^b^	Ref	20.83 (20–21.28)	23.26 (21.74–25)	Ref	16.39 (15.87–16.95)	17.86 (16.67–18.87)
Diabetes^b^	Ref	3.82 (3.40–4.29)	1.65 (1.01–2.69)	Ref	1.87 (1.66–2.11)	0.90 (0.55–1.47)
Stroke	Ref	4.81 (3.73–6.21)	11.11 (6.99–17.54)	Ref	4.17 (3.21–5.41)	9.71 (6.10–15.38)
Acute coronary syndrome	Ref	4.59 (2.63–8.00)	11.24 (4.15–30.30)	Ref	2.46 (1.45–4.18)	7.19 (2.92–17.86)
Heart failure	Ref	14.71 (11.49–18.87)	14.71 (7.87–27.78)	Ref	9.71 (7.94–11.90)	12.5 (8.06–19.23)
Venous thromboembolism	Ref	3.02 (2.51–3.64)	4.81 (3.13–7.35)	Ref	1.87 (1.61–2.18)	3.46 (2.51–4.76)
Pulmonary embolism	Ref	3.02 (2.46–3.72)	5.85 (3.80–9.01)	Ref	2.40 (1.94–2.96)	4.69 (3.05–7.25)
Death	Ref	2.28 (1,57–3,30)	5,62 (2,91–10,87)	Ref	1.72 (1.17–2.53)	4.83 (2.49–9.43)
MACE	Ref	5.0 (4.27–5.81)	8.0 (5.75–11.11)	Ref	3.91 (3.32–4.59)	6.90 (4.98–9.62)
CKD	Ref	21.74 (17.24–27.78)	47.62 (32.26–71.43)	Ref	10.87 (8.26–14.29)	29.41 (19.61–45.45)
CKD or dialysis^c^	Ref	23.26 (18.18–30.3)	50.0 (32.26–76.92)	Ref	10.31 (7.75–13.70)	26.32 (17.24–41.67)
Dialysis^c^	Ref	14.71 (7.41–29.41)	24.39 (5.75–100)	Ref	4.72 (2.20–10.20)	8.70 (2.02–37.04)

CKD, chronic kidney disease; HELLP, hemolysis, elevated liver enzymes, and low platelets syndrome; MACE, major adverse cardiovascular event (i.e., stroke, acute coronary syndrome, heart failure, or death); PE, preeclampsia.

a These models were adjusted on tobacco smoking, obesity, social deprivation and history of diabetes.

b The models estimating the hazard ratios of developing hypertension and diabetes were performed in the subpopulations of women without history of hypertension (*n* = 2,786,612) or diabetes (*n* = 2,815,058), respectively.

c At least 40 dialyses.

### Major Outcomes After the First Year Following Pregnancy

After the first year following pregnancy, as compared with controls, iPE was significantly associated with all outcomes, although these associations were weaker than during the first year following pregnancy (Table 5). In adjusted models, women who had iPE were more likely to develop chronic hypertension (aHR = 2.64 [2.56–2.73]), diabetes (aHR = 1.69 [1.59–1.80]), CKD (aHR = 3.97 [3.37–4.69]) and to have a MACE (aHR= 1.82 [1.65–2.01]) after the year following pregnancy, compared with controls. Women who had PE-HELLP were more likely to develop chronic hypertension (aHR = 1.91 [1.71–2.13]), CKD (aHR = 2.53 [1.35–4.72]), or start chronic dialysis (aHR = 5.00 [1.82–13.70]) after the year following pregnancy as compared with controls; however, the risk of other major adverse events was not significantly elevated (Table 5, Figure 2). The risk of chronic hypertension was greater in women with iPE than PE-HELLP, whereas the risk of CKD and chronic dialysis was not different in these 2 groups.

**Table 5:** Crude and adjusted Hazard Ratios of developing hypertension, diabetes or cardiovascular diseases after the 1st year following preeclampsia with or without HELLP syndrome

Parameters	Crude hazard ratios			Adjusted hazard ratios		
	No PE	PE without HELLP	PE-HELLP	No PE	PE without HELLP	PE-HELLP
Hypertension	Ref	2.87 (2.77–2.96)	1.96 (1.76–2.19)	Ref	2.64 (2.56–2.73)	1.91 (1.71–2.13)
Diabetes	Ref	2.64 (2.48–2.80)	1.24 (0.97–1.58)	Ref	1.69 (1.59–1.80)	0.92 (0.72–1.17)
Stroke	Ref	2.45 (2.11–2.87)	1.30 (0.72–2.36)	Ref	1.98 (1.69–2.32)	1.16 (0.64–2.10)
Acute coronary syndrome	Ref	3.14 (2.58–3.82)	1.72 (0.82–3.61)	Ref	2.16 (1.76–2.66)	1.50 (0.71–3.16)
Heart failure	Ref	4.74 (3.69–6.10)	2.78 (1.15–6.71)	Ref	2.55 (1.96–3.33)	1.99 (0.82–4.81)
Venous thromboembolism	Ref	1.80 (1.55–2.08)	1.47 (0.92–2.33)	Ref	1.44 (1.23–1.67)	1.31 (0.82–2.08)
Pulmonary embolism	Ref	1.63 (1.33–1.99)	1.12 (0.56–2.24)	Ref	1.29 (1.05–1.59)	0.99 (0.49–1.99)
Death	Ref	1.61 (1.34–1.93)	1.46 (0.84–2.51)	Ref	1.32 (1.10–1.60)	1.34 (0.78–2.32)
MACE	Ref	2.34 (2.12–2.58)	1.49 (1.06–2.10)	Ref	1.82 (1.65–2.01)	1.33 (0.94–1.87)
Chronic kidney disease	Ref	8.40 (7.25–9.80)	3.37 (1.81–6.29)	Ref	3.97 (3.37–4.69)	2.53 (1.35–4.72)
CKD or dialysis^a^	Ref	8.40 (7.25–9.8)	3.37 (1.81–6.29)	Ref	3.97 (3.37–4.69)	2.53 (1.35–4.72)
Dialysis^a^	Ref	18.52 (13.16–25.64)	10.64 (3.89–28.57)	Ref	5.85 (4.07–8.40)	5.00 (1.82–13.70)

CKD, chronic kidney disease; HELLP, hemolysis, elevated liver enzymes, and low platelets syndrome; MACE, major adverse cardiovascular event (i.e., stroke, acute coronary syndrome, heart failure of death); PE, preeclampsia.

In all models, the exposure to preeclampsia and HELLP syndrome was treated as a time-varying variable, consequently a woman could count in different categories across her follow-up.

The models estimating the hazard ratios of developing hypertension and diabetes were performed in the subpopulations of women without history of hypertension (*n* = 2,786,612) or diabetes (*n* = 2,815,058), respectively.

All models were adjusted on tobacco smoking, obesity, social deprivation and history of diabetes.

a At least 40 dialyses.

## Discussion

In the present study, we observed that PE with or without HELLP was associated with a major risk of hypertension, MACE, stroke, and death; and especially for CKD and chronic dialysis as compared with pregnancy without PE. Women who had PE-HELLP had a higher risk of cardiovascular events within the first year following pregnancy than women with PE without HELLP. The renal risk was particularly marked regarding the risk of CKD and dialysis within the first year. Our data indicated that dynamics of renal risk differed when HELLP is superimposed with PE versus when PE was isolated.

PE, particularly when associated with HELLP, was a strong predictor of CKD and dialysis. After adjustment, the risk of CKD or dialysis was increased by 2.5-fold and 5-fold, respectively, after the first year of follow-up. In contrast, a previous study with limited sample size found no increased long-term risk of CKD in women with prior HELLP compared with women who remained normotensive during pregnancy,^9^ a finding that is rather unique in the literature. In a recent UK study (2.5 million person-years of follow-up, 811 women with PE), the adjusted odds ratio for CKD was 1.96 (95% confidence interval: 1.45–2.56) compared with women who remained normotensive during pregnancy.^10^ Notably, no difference in CKD risk was observed between women with gestational hypertension and those with PE in this report.^10^ In a German cohort, PE was associated with similar CKD risks compared with normotensive pregnancies within a mean follow-up of 5.4 years.^11^ However, the risk of CKD was much higher in cases of preterm delivery.^11^ This association was confirmed in a nationwide cohort study from Denmark.^12^ In a Swedish registry-based cohort study, a similar risk of CKD was reported among women with PE (HELLP was included within the definition of PE) over a 20.7 years follow-up period of observation.^13^ None of these studies assessed the risk of CKD associated with HELLP either isolated or superimposed on PE.^10^, ^11^, ^12^, ^13^, ^14^, ^15^, ^16^ As early as 2008, PE during the first pregnancy was associated with a 5-fold relative risk of RF *vs* normal pregnancies in a study using the Norwegian National Renal Registry.^17^ Our own data from the CONCEPTION cohort study in France further indicated that recurrence of PE was associated with a much higher risk of CKD compared with normal pregnancies.^18^ These findings have been confirmed in more recent studies which sometimes combined PE and HELLP.^19^, ^20^, ^21^

However, the most remarkable finding was that the risk of CKD within the first year after delivery increased by 10.9-fold in patients with iPE and by 29.4-fold in patients with both PE and HELLP. The respective figures for RF were 4.7 and 8.7. These results demonstrate that HELLP superimposed on PE represented an additional major renal risk. In a study from the UK, the risk of CKD nearly doubled in women who experienced PE compared with those with normal pregnancies over a period of 6 to 8 weeks after delivery.^6^ However, the risk associated with HELLP was not assessed.^6^ To our knowledge, similar analyses have not been previously performed in the literature.

Overall, our findings indicate that the presence of HELLP confers a major risk of stroke, venous thromboembolism, pulmonary embolism, CKD, and dialysis as compared with women with iPE during the first year of follow-up. The real issue is whether HELLP should be considered as a complication of PE (and therefore, that the prognosis of women with HELLP and PE is understandably worse than that of women with only PE) or whether PE and HELLP represent distinct entities. Our data cannot resolve this issue; however, they provide new insights because the dynamics of the cardiovascular, and even more so, renal outcomes differed between HELLP and PE. PE and HELLP are usually considered similar entities. For practical reasons, because the incidence of HELLP is much lower than the incidence of PE, studies focused on isolated HELLP are usually underpowered: studying PE and HELLP together is easier, and it is usually done. Lower prevalence of hypertension, proteinuria, thrombophilia, and obesity was observed in patients with early HELLP as compared with those with early PE.^22^ Interestingly, the risk of stroke was greater in women with both PE and HELLP than in those with PE without HELLP. This finding is surprising because blood pressure elevation plays a major role in stroke development and is much more frequent in PE than in HELLP, indicating that the risk factors for stroke in patients with HELLP may be different.^23^ Moreover, dysregulation of the complement alternative pathway is implicated mainly in HELLP and much less in most cases of PE.^23^

This study has several strengths. First, its prospective and nationwide design enabled the inclusion of all hospital deliveries in France over a 10-year period, reducing selection bias and providing substantial statistical power to analyze the association between HELLP syndrome, a rare hypertensive disorder during pregnancy, and cardiovascular and renal outcomes, both of which are infrequent in young women. Lastly, because of the nature of the SNDS database, no participants were lost to follow-up throughout the study.

However, the study has some limitations. We identified chronic and gestational hypertension based on the dispensing of antihypertensive medications and diagnostic codes. As a result, some cases of untreated or undiagnosed gestational hypertension, particularly those that were mild, may have been overlooked, leading to an underestimation of associated risks. In addition, CKD identification may have been incomplete, because we only captured cases requiring hospitalization or dialysis. Censoring follow-up at the onset of subsequent pregnancies was necessary to avoid classification bias, because women not exposed to PE in their first pregnancy might develop it in a later one. However, this approach may have introduced bias, because women with HELLP or isolated PE are less likely to have additional pregnancies and thus less likely to be censored, potentially underestimating their long-term risks.^24^ Despite this, the lack of complete event identification is unlikely to be differentially affected by the presence or absence of hypertensive disorders and should not have introduced bias into the observed associations. Other limitations include a possible underestimation of CKD, with only more severe cases being captured, the fact that CKD coding during hospitalization may reflect advanced CKD and a possible misclassification bias for HELLP syndrome (i.e., diagnostic coding was not supported by biological data). Lastly, variables such as obesity and smoking were incompletely identified in our database, and we cannot fully exclude the potential for residual confounding.

In conclusion, this study provides new insights into risk factors and progression of major complications associated with iPE and PE-HELLP syndrome. Whether HELLP and PE represent separate or similar entities remains uncertain. These results highlight the critical importance of close monitoring and appropriate long-term follow-up with women with a history of HELLP syndrome, particularly with regard to renal function, in order to optimize management and reduce the risk of adverse outcomes.

## Disclosure

All the authors declared no competing interests.

## Author Contributions

J-MH, GL, J-BdF, and VO contributed to study conception, statistical analysis, writing of the first draft, and access to the data. LF, VM, J-BdF, and BS contributed to corrections of the drafts and interpretation of data. All the authors contributed to drafting and correction of the manuscript.

## Supplementary Material

Supplementary File (PDF) **Table S1.** ICD-10 codes used for the identification of exposures and outcomes.

## Footnotes

Supplementary File (PDF)
**Table S1.** ICD-10 codes used for the identification of exposures and outcomes.
