# Supplementary material for: Divergent Risk Factors and Outcomes in Hemolysis, Elevated Liver Enzymes, and Low Platelets Syndrome and Isolated Preeclampsia
Source: Kidney Int Rep. 2026 Jun 22;11(9):106681. doi: 10.1016/j.ekir.2026.106681 (PMC13400769; doi:10.1016/j.ekir.2026.106681)
Supplement: Supplementary File (PDF) — Table S1. ICD-10 codes used for the identification of exposures and outcomes. [file mmc1.pdf]

Supplemental Table S1: ICD-10 codes used for the identification of exposures and outcomes

-	ICD-10 codes	
	Primary diagnosis	Accompanying diagnosis
Exposures		
Pre-eclampsia	O11, O14, O15	
HELLP syndrome	O14.2	
Outcomes		
Stroke	I60, I61, I62, I63, I64, O225, O873	
Acute coronary syndrome	I200, I21, I22, I23	
Peripheral arterial disease	I702, I739, I740, I743, I744, I745	
Heart failure	I50, I11, I130, I132	
Heart failure (other identification)*	I139, J81, K761	I50, I11, I130, I132
Rhythm or conduction disorder	I44, I45, I47, I48, I49	
Pulmonary embolism	I26, O882	
Chronic kidney disease	N18, I12, I13, E102, E112, I122, I132,E142, Z490, Z940, N118, N1119	

*Heart failure was identified by a code of heart failure either as primary diagnosis, or as accompanying diagnosis when associated with a primary diagnosis of pulmonary oedema, liver congestion or hypertensive heart disease.
